# Stability of Dihydroartemisinin–Piperaquine Tablet Halves During Prolonged Storage Under Tropical Conditions

**DOI:** 10.4269/ajtmh.16-0759

**Published:** 2017-02-08

**Authors:** Eva Maria Hodel, Harparkash Kaur, Dianne J. Terlouw

**Affiliations:** 1 Liverpool School of Tropical Medicine, Liverpool, United Kingdom.; 2 Malawi-Liverpool-Wellcome Trust Clinical Research Programme, Blantyre, Malawi.; 3 London School of Hygiene and Tropical Medicine, London, United Kingdom.

## Abstract

Dihydroartemisinin–piperaquine (DP) is recommended for the treatment of uncomplicated malaria, used in efforts to contain artemisinin resistance, and increasingly considered for mass drug administration. Because of the narrow therapeutic dose range and available tablet strengths, the manufacturers and World Health Organization recommended regimens involve breaking tablets into halves to accurately dose children according to body weight. Use of tablet fractions in programmatic settings under tropical conditions requires a highly stable product; however, the stability of DP tablet fractions is unknown. We aged full and half DP (Eurartesim^®^) tablets in a stability chamber at 30°C and 70% humidity level. The active pharmaceutical ingredients dihydroartemisinin and piperaquine remained at ≥ 95% over the 3 months' period of ageing in light and darkness. These findings are reassuring for DP, but highlight the need to assess drug stability under real-life settings during the drug development process, particularly for key drugs of global disease control programs.

The antimalarial artemisinin-based combination therapy dihydroartemisinin–piperaquine (DP) is highly efficacious against uncomplicated malaria. Because of its long half-life and associated chemoprophylactic effect, DP is considered the most promising antimalarial for mass drug administration (MDA) for transmission reduction efforts in Africa.^1^ Furthermore, it is extensively studied for intermittent preventive treatment in children^2^,^3^ and pregnant women.^4^,^5^ The global discussion around use of drugs to reduce transmission has largely focused on DP's pharmacokinetic and pharmacodynamic properties, and those of suitable gametocytocidal drugs, whereas more practical threats to successful implementation have received much less attention.

Eurartesim (Sigma-Tau Industrie Farmaceutiche Riunite s.p.a., Rome, Italy), the only available fixed-dose combination of DP that has obtained marketing authorization from a stringent regulatory authority (i.e., the European Medicines Agency, [EMA]), is available as pediatric tablets containing 20 mg dihydroartemisinin and 160 mg piperaquine, and adult tablets containing 40 mg dihydroartemisinin and 320 mg piperaquine.^6^ Due to the narrow therapeutic dose range and limited number of tablet strengths, the manufacturer^6^ and the World Health Organization (WHO)^7^ recommend breaking tablets into halves to accurately dosing children according to their body weight. There is no WHO guidance on age-based dose regimens, where the use of half tablets may help limit the substantial increase in dose intake variation.^8^

The major concern with tablet fractions, apart from inaccurate dosing resulting from imprecise breaking of tablets, is the intrinsic stability of broken tablets of DP under conditions of high temperature and humidity found in malaria-endemic countries. Drug decomposition and formation of degradation products are typically accelerated in the presence of oxygen, moisture, heat, and strong light. As a consequence, remaining tablet halves may contain lower dose and, in the worst case, even toxic byproducts, potentially resulting in lack of clinical response, adverse events, and spread of antimalarial resistance. No data are available from the manufacturer, EMA registration, or scientific literature on the stability of DP tablet fractions once removed from the blister pack. In this study, we determined the stability of half tablets of DP at 37°C and 70% humidity as found in malaria-endemic countries.^9^ The results address the gap in knowledge on the stability of tablet halves of DP and this crucial information will help the successful implementation of MDA programs using DP.

Eurartesim (40/320 mg) tablets (*N* = 360; Lot 141450; Exp 11/2016) were purchased from Manson's Chemists Ltd (London E78BA, United Kingdom). Other chemicals were of high-performance liquid chromatography (HPLC) or analytical grade from various commercial sources.

The short-term stability of DP tablets halves was investigated within their expiry date (January to April 2016) in a “natural ageing” study over 3 months' light on (320–400 nm) and light off in climatic zone IV^9^ (30°C and 70% relative humidity) using a pharmaceutical stability chambers (PSC022, Weiss Gallenkamp, United Kingdom). Two hundred and forty tablets were manually split in half using as fragmentation of tablets was rather high when using a pill cutter. The halves were then put back in their blister. Supplemental Figures 1–7 show the photos of tablets being broken manually and placed in the blister pack for ageing. Two hundred and forty halves were kept in light and 240 halves in darkness in the stability chamber. At defined time points, that is, 0 hours (= nonaged), 24 hours, 3 days, 1 calendar month, and 3 calendar months, 48 tablet halves from each light exposure group were removed (Table 1), and stored in their blister packs at 4°C until analysis (within 1 week of removal from the chamber). One hundred and twenty full tablets were used as controls (Table 1).

Quantitative analysis of dihydroartemisinin and piperaquine was performed using a previously published HPLC with photo diode array detection method.^10^ Each full tablet or half tablet sample was dissolved in methanol first to obtain a 5 mg/mL solution of dihydroartemisinin. The sample was sonicated (10 minutes), 120 μL removed, and centrifuged. The supernatant (10 μL) was injected into the HPLC column and the amount of dihydroartemisinin present in the tablet was determined. To the rest of the solution 2 M HCl in methanol was added to obtain a 32 mg/mL stock solution of piperaquine, which was further diluted to get a 0.6 mg/mL dilute which was injected (20 μL) onto the HPLC column. HPLC analysis was conducted using a Dionex Ultimate 3000 system (Thermofisher, Hemel Hempstead, UK) and for the analysis of piperaquine, separation achieved using a Acclaim 120, C18, 5 μm Analytical (4.6 × 150 mm, Fisher Scientific, Leicestershire, UK). The mobile phase was a gradient of ammonium formate (10 mM, pH 2.7) and acetonitrile (v/v; 15:85 to 85:15 over 7.0 minutes). The separation of dihydroartemisinin was achieved using a GENESIS AQ 4 μm column (150 × 4.6 mm, Grace Materials Technologies, Cranforth, UK). The mobile phase was a gradient of ammonium formate (10 mM, pH 2.7) and acetonitrile (v/v; 30:70 over 5.0 minutes). The photodiode array detector (UV-PDA; DAD 3000) was set at 204 nm for dihydroartemisinin and 360 nm for piperaquine.

We tested the stability of Eurartesim full tablets and tablet halves when exposed to light or darkness at 30°C and 70% relative humidity for up to 3 months. The weights of tablets manually broken in half show the accuracy of this process (Table 2) and both, dihydroartemisinin and piperaquine amounts, remain fairly constant over 3 months in each group (Table 3). This addresses a key gap in implementation research, and reassures national malaria control programmes considering using DP for MDA as this will ensure the reduction of cost and wastage of unused tablet fractions.

The International Conference on Harmonization and the WHO state that to properly assess long-term stability of finished pharmaceutical products, testing on how the quality of the product varies with time under the influence of temperature, humidity, and light should be conducted under storage conditions experienced in the intended market. A recent study found that full tablets of Coartem^®^ (Novartis Pharma AG, Basel, Switzerland) (artemether–lumefantrine) and ASAQ Winthrop^®^ (Sanofi-Aventis, Gentilly, France) (artesunate–amodiaqiuine) were stable when “naturally aged” under tropical conditions. Acceptable levels of all active pharmaceutical ingredients (90–110% as per international pharmacopeia tolerance limits) were measured over 3 years, despite drugs having reached their expiry dates within 18–24 months from the start of the study.^11^

Information on the stability of tablet fractions is crucial especially when the number of available tablet strengths is limited to accurately dose all patient groups. In the case of DP, the manufacturer^6^ and the WHO^7^ recommend dosing regimen that, with the only available fixed-dose combination that has obtained marketing authorization from a stringent regulatory authority, can only be achieved by breaking tablets into halve. Although for individual treatment, caregivers can be advised to discard unused tablet fractions immediately after having administered the dose to the child to ensure no substandard tablets are administered during subsequent dosing time points, adherence to this strategy is not well known and seems even less feasible in an MDA setting where large quantities of wastage would be produced (with environmental and economic consequences). More information on stability of tablet fractions is therefore crucial for successful implementation of DP in programmatic settings as the knowledge base for drugs degraded as a result of storage in tropical climates is sparse at best^12^–^14^ and nonexistent for half tablets.

Findings from our study are product specific and cannot be extrapolated to other DP brands or formulations.

Information on stability of tablet fractions is crucial for successful implementation in programmatic settings, especially for drugs involving dosing recommendations based on tablet fractions in the absence of a child-friendly formulation. We recommend this type of information be collected much earlier in the drug development process and clearly stated on the package insert.

## Supplementary Material

Supplemental figures.

## Figures and Tables

**Table 1:**
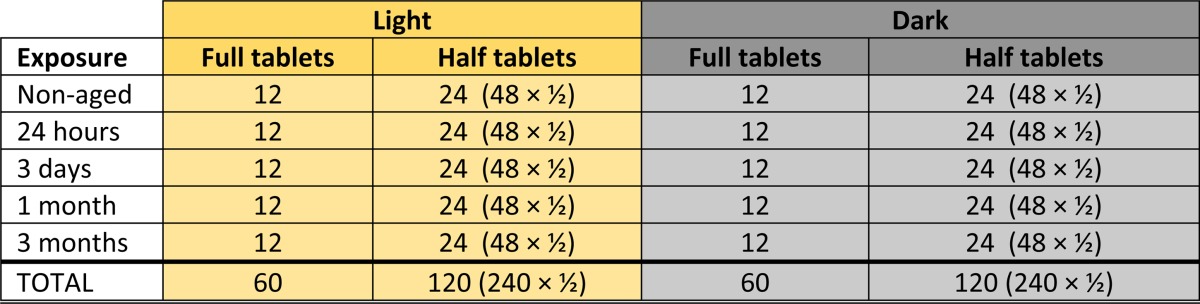
Number of tablets analyzed

**Table 2:**
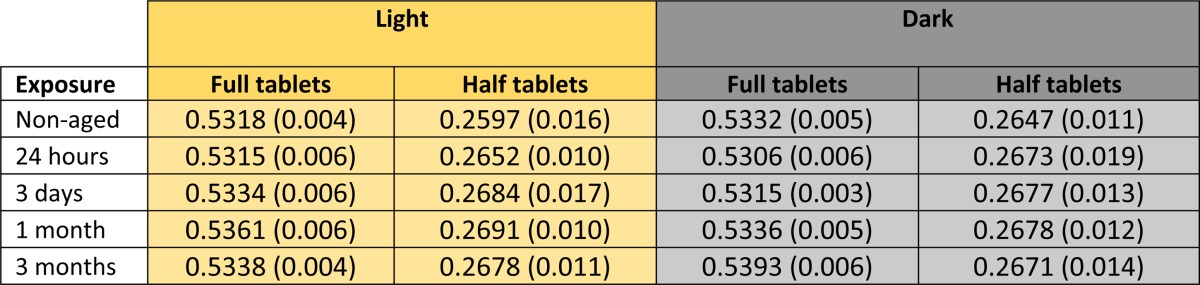
Weight of tablets analyzed

Mean weight in grams (standard deviation) for the numbers of tablets and tablet halves specified in Table 1.

**Table 3:**
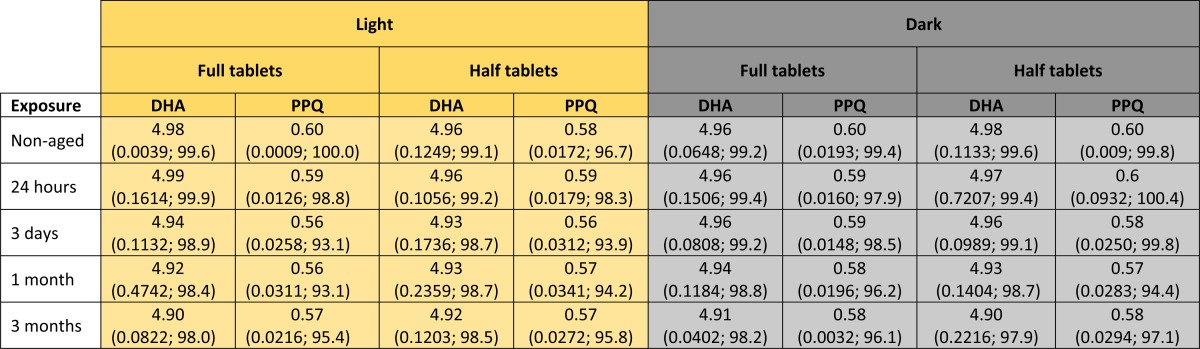
Amounts of dihydroartemisinin (DHA) and piperaquine (PPQ) measured over time

Mean amount in mg/mL (SD; % active pharmaceutical ingredient) for the numbers of tablets and tablet halves specified in Table 1.
