# Supplementary material for: Stability of Dihydroartemisinin–Piperaquine Tablet Halves During Prolonged Storage Under Tropical Conditions
Source: Am J Trop Med Hyg. 2017 Feb 8;96(2):338–40. doi: 10.4269/ajtmh.16-0759 (PMC5303033; doi:10.4269/ajtmh.16-0759)
Supplement: Supplementary file 1 [file SD4.pdf]

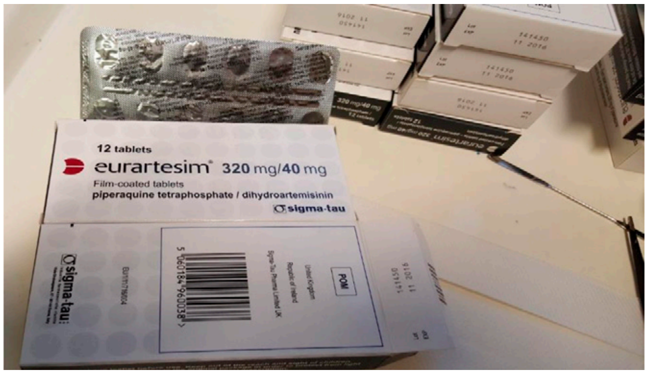

Supplemental Figure 1. Packets of Eurartesim 320 mg/40 mg used in the study.

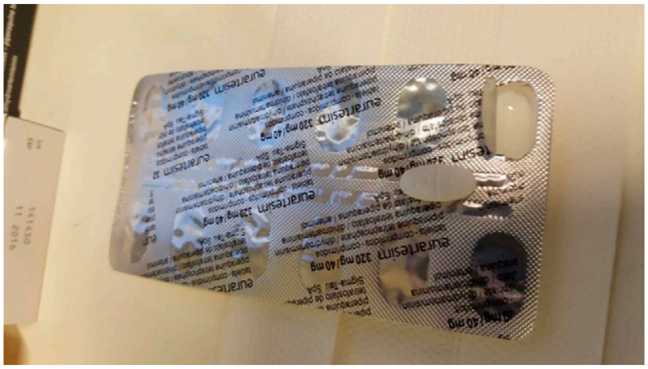

Supplemental Figure 2. Blister showing a tablet removed.

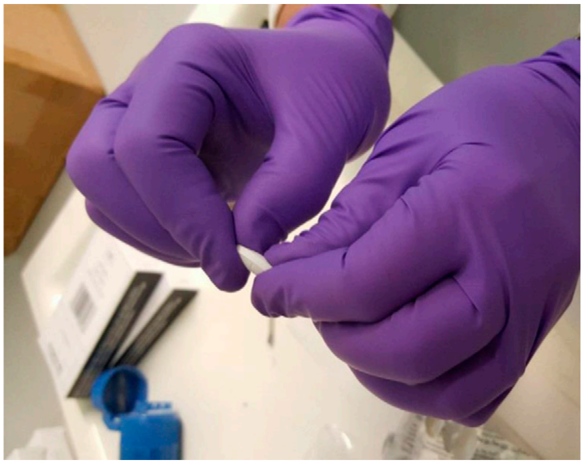

Supplemental Figure 3. Manual tablet breaking.

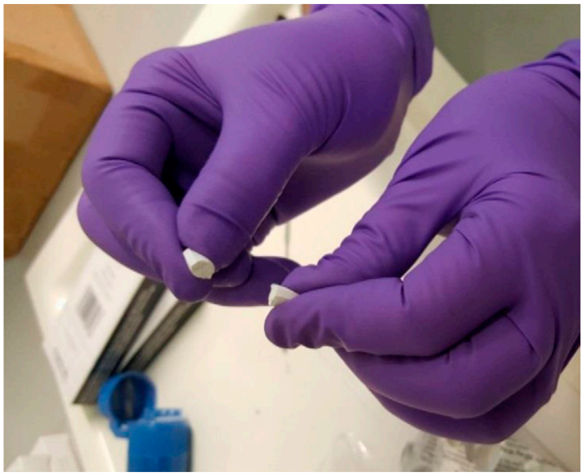

Supplemental Figure 4. Two tablet halves.

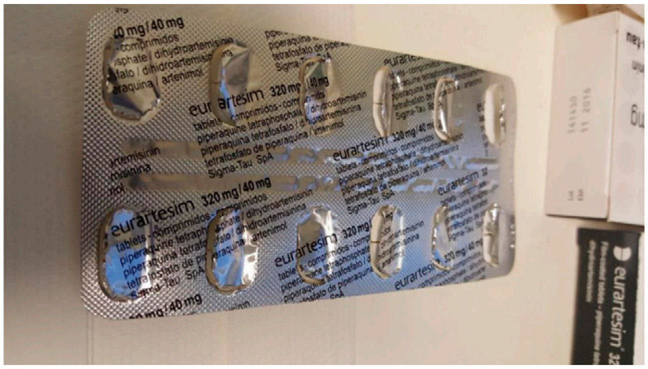

Supplemental Figure 5. Tablet halves placed back in the blister.

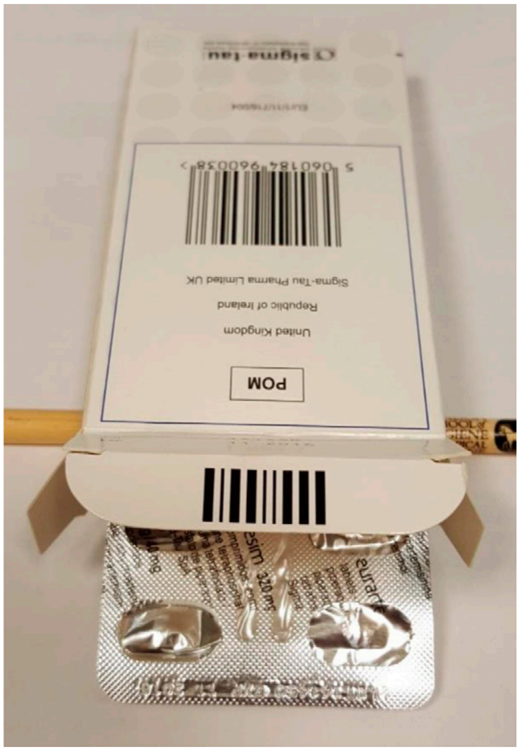

Supplemental Figure 6. Blister back in the packet with the tablet halves ready for ageing.

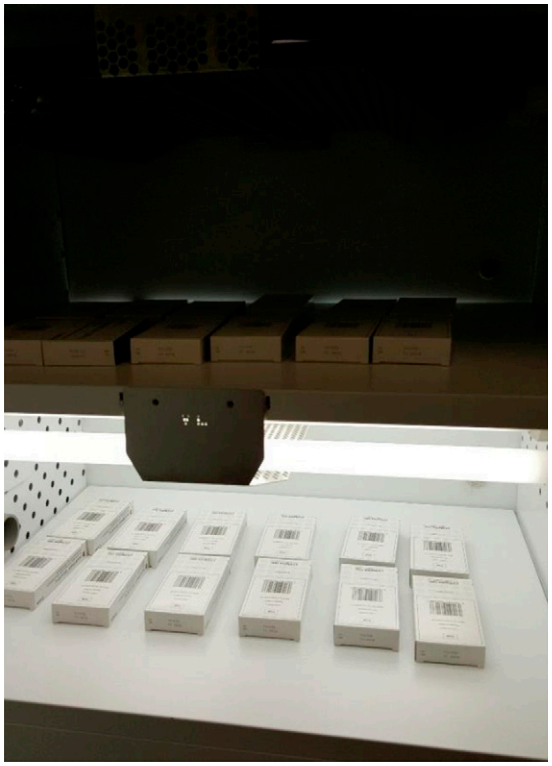

Supplemental Figure 7. Boxes of full and half tablets in the stability chamber in presence (bottom) and absence (top) of light.
